# Antibody Secretion Capacity in CVID Patients: Immunoglobulin Isotypes and Antigen Specificities After T-Cell-Dependent In Vitro Stimulation †

**DOI:** 10.3390/jcm14207246

**Published:** 2025-10-14

**Authors:** Sophie Steiner, Kirsten Wittke, Sandra Bauer, Carmen Scheibenbogen, Leif G. Hanitsch

**Affiliations:** 1 Institute of Medical Immunology, Charité-Universitätsmedizin Berlin, Corporate Member of Freie Universität Berlin and Humboldt Universität zu Berlin, Augustenburger Platz 1, 13353 Berlin, Germany; 2 Center for Regenerative Therapies (BCRT), Berlin Institute of Health (BIH), Charité-Universitätsmedizin Berlin, Charitéplatz 1, 10117 Berlin, Germany

**Keywords:** common variable immunodeficiency disorder (CVID), inborn error of immunity (IEI), antibody-secreting cells (ASCs), memory B-cell differentiation, ELISpot

## Abstract

**Background:** Common variable immunodeficiency (CVID), the most prevalent symptomatic inborn error of immunity, involves impaired B-cell differentiation and antibody production, causing recurrent infections and the need for life-long immunoglobulin replacement therapy. **Methods:** This study evaluated the *in vitro* differentiation of memory B-cells (MBCs) into antibody-secreting cells (ASCs) in CVID patients. Peripheral blood mononuclear cells from 13 CVID patients and 10 healthy controls were stimulated using two protocols: (I) Staphylococcus aureus Cowan Strain I, Pokeweed mitogen, and CpG, or (II) a T-cell-dependent approach using CD40 ligand, interleukin-21, and CpG. B-cell subpopulations were analyzed by flow cytometry, ASC differentiation using ELISpot, and antibody levels in supernatants by ELISA. **Results:** Despite severely restricted *in vivo* antibody production, MBCs from all 13 CVID patients differentiated into IgG and IgM ASCs under adequate *in vitro* stimulation. Protocol II, mimicking T-cell help, was more effective than protocol I. As expected, the patients exhibited reduced class-switched MBCs *ex vivo*, but the MBCs differentiated and proliferated to an extent similar to those in healthy controls. IgA secretion remained significantly impaired post-stimulation. Specific IgG antibodies against tetanus toxoid and *Streptococcus pneumoniae* were detected in the patient supernatants, while no double-stranded DNA autoantibodies emerged after *in vitro* stimulation. **Conclusions:** These findings indicate that the MBCs of most patients retain functional B-cell differentiation and antigen-specific IgG secretion under T-cell dependent stimulation, though IgA secretion remains impaired. Tailored stimulation protocols could deepen our understanding of how to restore MBC formation in CVID patients *in vivo*. This methodology provides a platform to investigate antigen-specific functional memory responses like post-vaccination.

## 1. Introduction

Common variable immunodeficiency disorder (CVID) is the most prevalent clinically relevant inborn error of immunity (IEI), affecting approximately 1 in 25,000 individuals worldwide [1]. According to the criteria of the European Society for Immunodeficiencies (ESID), the defining immunological features of CVID include hypogammaglobulinemia (reduced IgG and IgA, with or without decreased IgM), qualitative antibody deficiency (impaired vaccine response), as well as functional and phenotypic abnormalities in B-cell subsets, in particular a severe reduction of class-switched (CS) memory B-cells (MBCs) and plasmablasts (PBs), which represent the main source of antibody-secreting cells (ASCs) [2,3]. This predisposes patients to severe and recurrent infections [4,5]. In addition, 30–50% of patients experience a range of non-infectious complications due to immune dysregulation such as granulomatous disorders and lymphoproliferation and show a slightly enhanced susceptibility to malignancies [6].

Genetic diagnostics using whole exome sequencing (WES) has contributed significantly to the pathophysiological understanding of CVID by identifying monogenetic causes in about 20% of patients [7]. However, precise immunological pathomechanisms remain unclear in the majority of patients.

In line with the heterogeneous clinical presentation, several immunological defects in B-cell activation, differentiation, and proliferation have been proposed in CVID [8], including defects in germinal center (GC) formation and function, affecting the processes of somatic hypermutation (SHM) and affinity maturation [9]. Furthermore, abnormal B-cell signaling pathways have been observed in some CVID patients involving the B-cell receptor (BCR) and co-stimulatory molecules like CD19 or CD21 [10]. Although initially regarded as a disease of the B-cell compartment, it soon became clear that abnormalities in T-cell function, impeding effective B-cell help, contribute to disease pathophysiology [11,12,13,14]. The broad spectrum of impairments in both B-cell and T-cell functions underscores the complexity of humoral immune deficiencies in CVID patients and highlights the need for targeted therapeutic strategies to restore effective B-cell responses.

Our understanding of *in vitro* B-cell differentiation in CVID patients is limited, constraining a comprehensive understanding of B-cell expansion capacity in this patient group. Following early work by Bryant et al., B-cell potential for the *in vitro* secretion of antibodies was used for CVID classification purposes [15]. Although subsequent studies indicated that CVID patients’ B-cells can respond to specific stimuli, their immunoglobulin production is predominantly characterized by IgM, often with reduced ability to produce IgG or IgA [16,17]. While reduced levels of CS MBCs are part of current CVID classifications [18], functional *in vitro* capacity of the remaining CS MBCs of CVID patients are poorly characterized [16] and data on the specificity of *in vitro* induced antibody production is missing.

In this study, we aimed to evaluate the *in vitro* differentiation capacity of MBCs of CVID patients by cultivating and stimulating peripheral blood mononuclear cells (PBMCs) using two different stimulation protocols, differing in their potential to mimic T-cell-dependent immune responses. We used flow cytometry to analyze B-cell subsets on day 0 (baseline) and day 7 (post-stimulation) of cell culture. Functional MBC response was further analyzed using an ELISpot assay with expanded cells on day 7 of cell culture to quantify the secretion of immunoglobulin classes IgG, IgA, and IgM. Memory ELISpot assays are a validated methodology for assessing the antibody secretion attained from reactivated MBCs [19]. The quality of the stimulation protocol and antibody responses was further analyzed by measuring cell culture supernatants using different ELISA assays to detect specific tetanus toxoid and pneumococcus antibodies, as well as double-stranded (ds)-DNA autoantibodies.

## 2. Materials and Methods

### 2.1. Blood Samples

CVID patient samples (n = 13) were collected at the outpatient clinic for immunodeficiencies in adults at Charité Universitätsmedizin Berlin, Germany. The diagnosis of CVID was based on ESID criteria [20]. The patients received continuous IgG replacement therapy. Healthy controls (HCs, n = 10), not suffering from acute or chronic infections, were recruited from laboratory staff. The study was approved by the Ethics Committee of Charité Universitätsmedizin Berlin in accordance with the 1964 Declaration of Helsinki and its later amendments (EA2-046-12). Written informed consent was obtained from all patients and HCs.

### 2.2. Isolation of Peripheral Blood Mononuclear Cells

PBMCs were isolated by density gradient centrifugation from heparinized whole blood using Pancoll separation medium (PAN-Biotech, Aidenbach, Germany) in Leucosep tubes (Greiner Bio-One, Kremsmünster, Austria). To maintain optimal cell viability and function, all blood samples were processed within a maximum of four hours after collection. Isolated PBMCs were directly used for *in vitro* cell culture and flow cytometric analysis.

### 2.3. In Vitro PBMC Cell Culture and Stimulation

**Figure 1:**
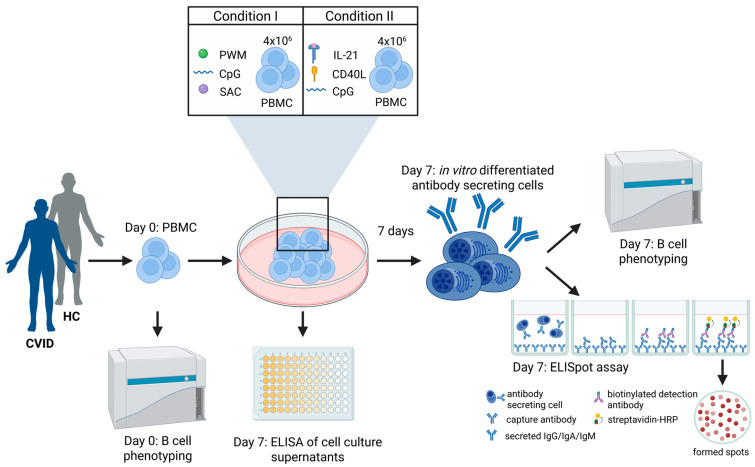
Experimental setup for comprehensive B-cell expansion studies. PBMC samples of CVID patients (n = 13) and HCs (n = 10) were stimulated for 7 days with condition I and II protocol for B-cell expansion. PBMCs on day 0 before stimulation and on day 7 after stimulation were analyzed by flow cytometry B-cell phenotyping. Functional memory B-cell (MBC) response of the expanded cells on day 7 was analyzed with an MBC ELISpot assay. Quantitative and qualitative antibody responses after *in vitro* stimulation were assessed in cell culture supernatants on day 7 using different commercially available ELISA kits. CD40L = CD40 ligand, CpG = CpG oligodeoxynucleotides, CVID = common variable immunodeficiency disorder, HRP = horseradish peroxidase, IL-21 = interleukin 21, PBMC = peripheral blood mononuclear cell, PWM = pokeweed mitogen, SAC = staphylococcus aureus Cowan I. Created in BioRender. Steiner, S. (2025) https://BioRender.com/gtrms1r.

In order to obtain the populations of interest (ASC) required for the ELISpot assay, freshly isolated PBMCs from CVID patients and HCs were stimulated for B-cell differentiation using two distinct protocols for a total duration of seven days (Figure 1). The cells were seeded in at a density of 4 × 10^6^ cells per 3 mL RPMI medium supplemented with 10% fetal calf serum (FCS) and 1% penicillin/streptomycin (culture medium) in a 6-well plate in the presence of 5% CO_2_ at 37 °C (standard settings). The first stimulation protocol (condition I) for B-cell differentiation was adapted from Crotty et al. and included 6 µg/mL CpG ODN 2006 (InvivoGen, San Diego, CA, USA), 100 ng/mL of Pokweed mitogen (PWM), *Staphylococcus aureus* Cowan Strain I (SAC; 1:10,000), and 50 μM β-Mercaptoethanol (β-ME; Sigma-Aldrich, St. Louis, MO, USA) [21]. The second B-cell stimulation protocol (condition II) involved 270 ng/mL CD40L (Biolegend, San Diego, CA, USA), 30 ng/mL IL-21 (ImmunoTools, Friesoythe, Germany), and 6 µg/mL CpG ODN 2006 (InvivoGen). Following the 7-day stimulation period, the cells were harvested and washed twice with culture medium prior to ELISpot analysis. Cell culture supernatants from day 7 were collected and stored at −80 °C until further use.

### 2.4. Memory B-Cell ELISpot Assay

To detect ASCs, an ELISpot assay was performed after the 7-day stimulation of PBMCs for B-cell differentiation under conditions I and II (Figure 1). Then, 96-well MultiScreen Filter Plates (Merck Millipore, Burlington, MA, USA) were coated with 1.2 μg/mL goat anti-human IgG, 15 μg/mL goat anti-human IgA, 10 μg/mL goat anti-human IgM (Jackson ImmunoResearch, West Grove, PA, USA), or phosphate-buffered saline (PBS; negative control to assess unspecific antibody binding) in the respective wells and incubated overnight at 4 °C. On the following day, the plates were blocked with culture medium for 1 h under standard settings, while the harvested cells of day 7 after the stimulation were washed twice with culture medium. The cells were plated in duplicate onto the ELISpot plate at dilutions of 12.5 × 10^3^ and 6.25 × 10^3^ cells per 100 μL culture medium per well and incubated for 4 h under standard settings. After incubation, the wells were washed six times with 1xPBS, 1% bovine serum albumin (BSA), and 0.05% Tween (washing buffer). Biotin-conjugated secondary antibodies were applied (100 μL/well; goat anti-human IgG-Biotin (1:5000, SouthernBiotech, Birmingham, AL, USA), anti-human IgA-Biotin (1:500, Invitrogen, Waltham, MA, USA), and anti-human IgM-Biotin (1:5000, SouthernBiotech)) and incubated ON at 4 °C. After incubation, the wells were washed six times with washing buffer followed by incubation with 2.5 μg/mL Streptavidin-HRP (Biolegend, San Diego, CA, USA) for 1 h at room temperature (RT). Additional washing was performed 3 times using 1 × PBS. Spot development was initiated by adding substrate buffer (352 mL 0.3 M sodium acetate solution, 148 mL 0.2 M acetic acid solution, 500 mL aqua dest., pH = 5.0) supplemented with 3-amino-9-ethyl-carbazole (AEC)-dimethylformamide (DMF) solution (1:30) and 3% H_2_O_2_ (1:100). The reaction was terminated by rinsing the plates with water. After complete drying of the wells, ELIspots were analyzed using an AID ELISpot reader system (AID GmbH, Penzberg, Germany).

### 2.5. Flow Cytometry B-Cell Phenotyping

FACS analysis of B-cell subsets was performed with PBMCs before stimulation on day 0 (*ex vivo*) and on day 7 after stimulation (*in vitro*) under conditions I and II (Figure 1). The cells were washed twice with 1xPBS and stained with a Live/Dead marker for 30 min at RT (Table S1). Subsequently, surface staining was performed for 30 min at 4 °C using monoclonal antibodies (Table S1). Acquisition was performed using a CytoflexLX flow cytometer (Beckman Coulter, Brea, CA, USA) and analyzed with FlowJo software version 10.0.8. EUROClass classification [18] was used for the gating strategy of the B-cell subsets (Table S2 and Figure S1).

### 2.6. Analysis of In Vitro Differentiation Capacity of Memory B-Cells into Antibody Secreting Cells

The MBC ELISpot assay was conducted to functionally characterize the ASCs derived from *in vitro* differentiated MBCs following the 7-day stimulation period. While the ELISpot assay alone quantifies ASCs per 1 × 10^6^ PBMCs, it does not account for the cellular composition of the cultured PBMC population, particularly the frequencies of relevant B-cell subpopulations that give rise to ASCs. To address a more detailed picture of the B-cell composition in CVID patients and HCs within the cultured PBMC population, flow cytometric analysis of B-cell subpopulations was performed before stimulation on day 0 *ex vivo* and on day 7 *in vitro* after stimulation. B-cell phenotyping followed the EUROclass classification system by Wehr et al. [18], allowing identification of key subsets on CD19+ B-cells, CS and IgM^+^ MBCs, as well as CS and IgM^+^ PBs. By integrating ELISpot results with flow cytometry quantification of the crucial B-cell subpopulations, the ASCs were normalized to a fixed number of cells, specifically per 10,000 CD19+ B-cells, 1000 CS and IgM^+^ MBCs, and PBs. This normalization approach addresses the substantial inter-individual variability in B-cell subset distribution, particularly in CVID patients, who often exhibit pronounced reductions in specific B-cell compartments.

### 2.7. ELISA of Cell Culture Supernatants

Cell culture supernatants of day 7 were analyzed for specific antibodies using ELISA (Figure 1). First, whole IgG, IgA, and IgM (Abnova, Taipei City, Taiwan) antibody levels were determined according to the manufacturer’s instructions. Following this, specific antibodies for anti-dsDNA IgG (EUROIMMUN Medizinische Labordiagnostika AG, Lübeck, Germany), anti-Tetanus Toxoid (T. Tox) IgG, and anti-pneumococcal capsular polysaccharide (PCP) IgG (both The Binding Site, Birmingham, UK) were performed based on the manufacturer’s guidelines.

### 2.8. Statistical Analyses

Statistical analyses were performed using GraphPad Prism version 10 (GraphPad Software, San Diego, CA, USA). The Kruskal–Wallis test, followed by Dunn’s post hoc test, was used for unpaired comparisons between multiple groups to find significant differences. If significance was detected, the two-tailed Mann–Whitney U test was applied for unpaired comparisons across the two groups, and paired comparisons within a group were assessed with the Wilcoxon signed-rank test. Continuous variables are presented as medians with interquartile ranges (IQRs). Statistical significance was defined as * *p* < 0.05, ** *p* < 0.01, *** *p* < 0.001, and **** *p* < 0.0001.

## 3. Results

### 3.1. Patient Cohort Characteristics

The study enrolled 13 CVID patients (female–male ratio of 7:6) fulfilling ESID criteria. The median age was 53 years. Detailed patient demographics and immunological and clinical characteristics are shown in Table 1 and Table 2. Genetic testing included WES by next-generation sequencing (NGS) and revealed a heterozygous variant of unknown significance (VUS) in PIK3CD (ACMG class III) in patient 4 and another heterozygous VUS in IRF2BP2 (ACMG class III); all other patients had negative results for WES. The HCs had a median age of 32 (18–63) and a female–male ratio of 4:6.

### 3.2. Phenotypic Flow Cytometry Data for B-Cell Differentiation

**Figure 2:**
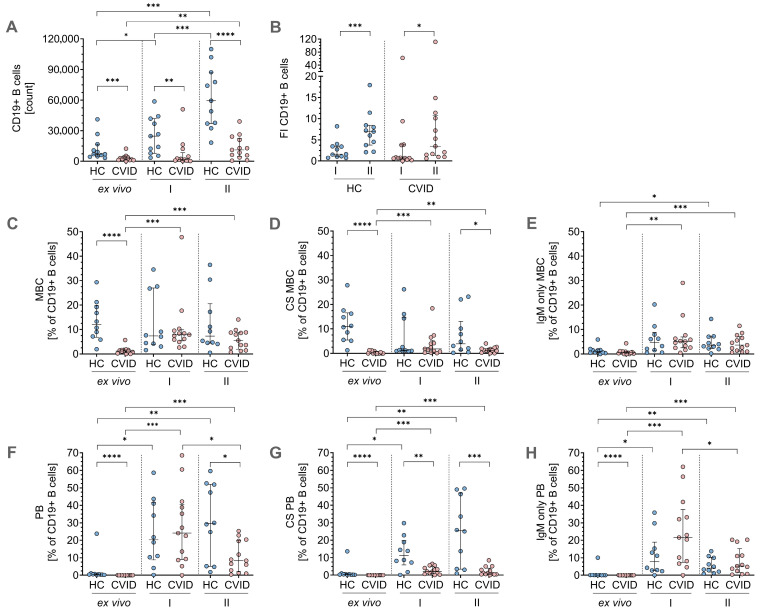
Comparison of B-cell count and frequencies of memory B-cells and plasmablasts *ex vivo* and after *in vitro* stimulation. B-cell subset analysis was performed using flow cytometry in healthy controls (HC) and patients with common variable immunodeficiency (CVID) before and after stimulation with protocol I and II. (**A**) Total CD19^+^ B-cell count *ex vivo* and after *in vitro* stimulation and (**B**) fold induction of CD19^+^ B-cell count after stimulation. (**C**) Frequencies of memory B-cells (MBCs), (**D**) class-switched (CS) MBCs, and (**E**) IgM-only MBCs *ex vivo* and after stimulation. (**F**) Frequencies of plasmablasts (PBs), (**G**) CS PBs, and (**H**) IgM-only PBs. Statistical analyses were performed using the Kruskal–Wallis test for group comparisons with Dunn’s post hoc test. Unpaired comparisons across the two groups were done by the two-tailed, non-parametric post hoc Mann–Whitney U test; paired comparisons within a group were done using the paired, non-parametric post hoc Wilcoxon matched pairs signed-rank test. Significance levels are indicated as *: *p* ≤ 0.05; **: *p* ≤ 0.01; ***: *p* ≤ 0.001; ****: *p* ≤ 0.0001.

To evaluate the proliferative capacity of the two B-cell stimulation protocols during *in vitro* culture, we first quantified the total number of CD19^+^ B-cells derived from 4 × 10^6^ PBMCs on day 0 of cell culture. Under condition I, a significant increase in the number of CD19^+^ B-cells compared to baseline levels in the HCs was observed, whereas in the CVID patients, B-cell numbers increased as well, but showed no significant difference compared to the *ex vivo* condition (Figure 2A). In contrast, condition II stimulation protocol led to significant *in vitro* B-cell expansion in the HCs and CVID patients (Figure 2A), indicating a superior stimulatory effect compared to condition I. However, absolute counts of CD19^+^ B-cells remained significantly lower in the CVID patients compared to HCs *ex vivo* at baseline and under both *in vitro* stimulation conditions (Figure 2A). When assessing fold induction (FI) of CD19^+^ B-cells relative to *ex vivo* levels, stimulation with condition II yielded substantially higher FI values in both HCs (FI = 7) and CVID patients (FI = 3.5). Condition I stimulation, on the other hand, resulted in modest expansion in the HCs (FI = 1.7), whereas in the CVID patients, protocol I resulted in overall fewer B-cells and hence lower FI (FI = 0.7; Figure 2B).

Regarding MBCs, the CVID patients had, as expected, lower initial *ex vivo* frequencies of IgD^−^CD27^+^ MBCs compared to the HCs. Both condition I and II stimulation resulted in increased frequencies of IgD^−^CD27^+^ MBCs in the CVID patients, while frequencies in the HCs remained similar (Figure 2C). Additionally, both stimulation protocols led to higher frequencies of CS MBCs in the CVID patients, but not in the HCs. Following condition II stimulation, CS MBC frequencies were significantly lower in the CVID patients than in the HCs (Figure 2D). Furthermore, IgM^+^ unswitched MBCs significantly increased in the CVID patients after condition I and II *in vitro* cell culture and in the HCs following protocol II stimulation (Figure 2E).

On day 0, *ex vivo* analysis of PBs revealed significantly lower frequencies of IgD^−^ PBs, IgM^−^ CS PBs, and IgM^+^ PBs in the CVID patients in comparison to the HCs (Figure 2F–H). Both *in vitro* stimulation protocols significantly increased the total frequencies of IgD^−^ PBs (Figure 2F), as well as CS PBs (Figure 2G) and IgM^+^ PBs (Figure 2H) in the HCs and CVID patients. Despite the observed increases in frequencies after stimulation, levels of CS PBs after condition I and II cell culture remained significantly lower in the CVID patients compared to the HCs (Figure 2G).

Taken together, the results from flow cytometry demonstrate that both protocols were able to generate the populations of interest, including CS MBCs and CS PBs, and showed that condition II stimulation protocol is more effective than condition I in enhancing *in vitro* expansion of CD19^+^ B-cells in HCs and CVID patients. However, despite increased frequencies after *in vitro* stimulation in CS MBCs (Figure 2D) and CS PBs (Figure 2G) in the CVID patients, they consistently exhibited lower levels compared to the HCs.

### 3.3. MBC ELISpot Assay: Immunoglobulin Secretion Capacity After In Vitro Stimulation and Differentiation

**Figure 3:**
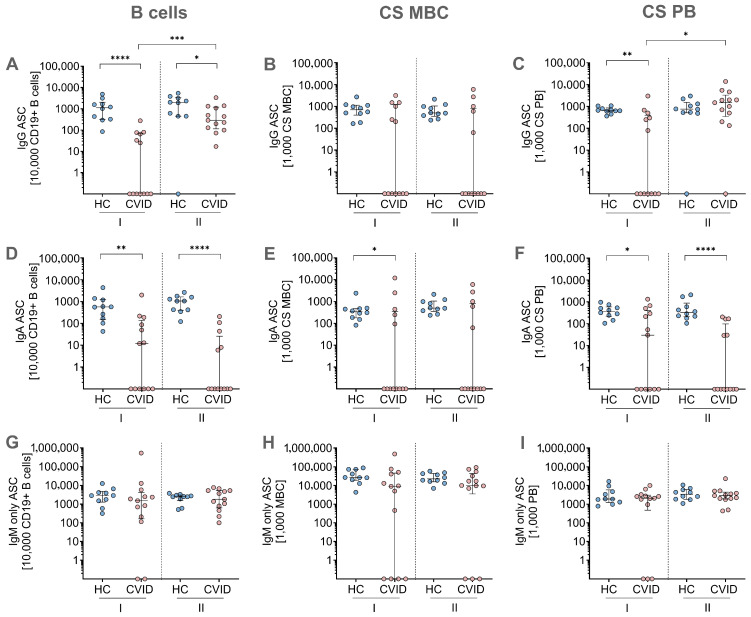
Results of the ELISpot assay measuring the secretion of IgG, IgA, and IgM after *in vitro* stimulation with protocols I and II in healthy controls (HCs) and patients with common variable immunodeficiency (CVID). (**A**) IgG-secreting CD19^+^ B-cells, (**B**) IgG-secreting class-switched memory B-cells (CS MBCs), (**C**) IgG-secreting class-switched plasmablasts (CS PBs), (**D**) IgA-secreting CD19^+^ B-cells, (**E**) IgA-secreting CS MBCs, (**F**) IgA-secreting CS PBs, (**G**) IgM-only secreting CD19^+^ B-cells, (**H**) IgM-only secreting MBCs, and (**I**) IgM-only secreting PBs. ASCs were calculated per 10,000 CD19^+^ B-cells, 1000 CS and IgM-only MBCs, and PBs to normalize inter-individual variability in B-cell subset distribution, especially present in CVID patients, allowing comparison of functional B-cell responses to HCs. Statistical analyses were performed using the Kruskal–Wallis test for group comparisons with Dunn’s post hoc test. Unpaired comparisons across the two groups were done by the two-tailed, non-parametric post hoc Mann–Whitney U test; paired comparisons within a group were done using the paired, non-parametric post-hoc Wilcoxon matched pairs signed-rank test. Significance levels are indicated as *: *p* ≤ 0.05; **: *p* ≤ 0.01; ***: *p* ≤ 0.001; ****: *p* ≤ 0.0001.

In parallel, MBC ELISpot experiments were conducted using *in vitro* stimulated cells of day 7 to detect IgG, IgA, and IgM ASCs (Figure 3). To complement the ELISpot data, the same PBMCs used in the assay were analyzed by flow cytometry *ex vivo* on day 0 and after *in vitro* stimulation on day 7 to phenotype distinct B-cell subpopulations. This subsequently allowed calculation of spots per 10,000 CD19^+^ B-cells and 1000 CS MBCs or CS PBs, as described in detail in the Section 2.

The MBC ELISpot assay revealed IgG responses in all HCs and 6 CVID patients following stimulation with protocol I for CD19^+^ B-cells, CS MBCs, and CS PBs (Figure 3A–C). Notably, IgG responses were induced in all CVID patients after condition II stimulation when calculated for CD19^+^ B-cells (Figure 3A) and in 12/13 CVID patients for CS PBs (Figure 3C), which are the primary source of ASCs. However, IgG responses were significantly lower in the CVID patients compared to the HCs for both stimulation protocols when calculated per CD19+ B-cells (Figure 3A). Within the CVID patients, CD19^+^ IgG responses were significantly higher after stimulation condition II compared to stimulation condition I (Figure 3A). Remarkably, stimulation condition II led to similar IgG responses per CS PBs in the CVID patients and HCs (Figure 3C). In CS MBCs, IgG ELISpot responses did not differ between both stimulation protocols (Figure 3B). However, fewer CVID patients (n = 5) had IgG responses per CS MBC, whereas all HCs were capable of mounting a response (Figure 3B).

IgA-secreting cells were detected by ELISpot in all HCs and in up to 7 CVID patients (Figure 3D–F). Overall, IgA spots were significantly lower in the CVID patients compared to the HCs as assessed per 10,000 CD19^+^ B-cells (Figure 3D), 1000 CS MBCs (Figure 3E), and 1000 CS PBs (Figure 3F). No significant differences in IgA secretion were observed between the two distinct stimulation conditions within the respective patient and control groups (Figure 3D–F).

In contrast, all CVID patients and HCs were able to secrete IgM in the ELISpot assay following stimulation condition II and up to 11 CVID patients after stimulation condition I (Figure 3G–I). IgM responses did not differ between the groups (HC and CVID) or between both stimulation conditions across all subsets analyzed, including spots per 10,000 CD19^+^ B-cells (Figure 3G), 1000 CS MBCs (Figure 3H), and 1000 CS PBs (Figure 3I).

### 3.4. In Vitro Stimulation Protocols Induced Antigen-Specific Immunoglobulin Secretion

**Figure 4:**
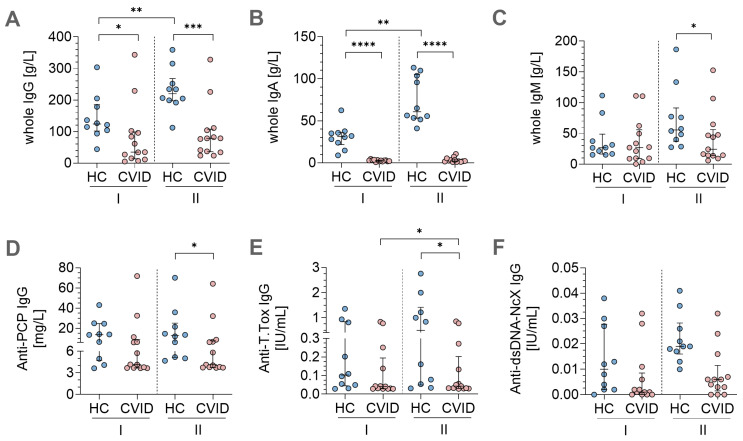
Immunoglobulin secretion in cell culture supernatants assessed by ELISA. Results of ELISA assays measuring whole immunoglobulin levels and antigen-specific IgGs in cell culture supernatants after *in vitro* stimulation with protocols I and II in healthy controls (HC) and patients with common variable immunodeficiency (CVID). (**A**) Whole IgG; (**B**) whole IgA; (**C**) whole IgM; (**D**) anti-pneumococcal capsular polysaccharide (PCP) IgG; (**E**) anti-Tetanus Toxoid (T. Tox) IgG; (**F**) anti-double-stranded (ds) DNA IgG. Statistical analyses were performed using the Kruskal–Wallis test for group comparisons with Dunn’s post hoc test. Unpaired comparisons across the two groups were done by the two-tailed, non-parametric post hoc Mann–Whitney U test; paired comparisons within a group were done using the paired, non-parametric post hoc Wilcoxon matched pairs signed-rank test. Significance levels are indicated as *: *p* ≤ 0.05; **: *p* ≤ 0.01; ***: *p* ≤ 0.001; ****: *p* ≤ 0.0001.

Cell culture media after 7 days of *in vitro* stimulation were analyzed for total IgG, IgA, and IgM to quantify antibody levels in supernatants using ELISA (Figure 4A–C). Overall, IgG levels (Figure 4A) were highest in the cell culture supernatants, followed by IgM (Figure 4C) and IgA (Figure 4B).

Total IgG, IgA, and IgM levels were significantly lower in the CVID patients compared to the HCs after both stimulation protocols (Figure 4A–B), with the exception of IgM levels following condition I stimulation, which were similar between the two groups (Figure 4C). Condition II stimulation induced significantly higher IgG, IgA, and IgM in the HCs. This effect was most pronounced in IgA responses (Figure 4B). Antibody levels in the CVID patients after stimulation II also increased, but not to a significant extent.

In the next step, we aimed to further evaluate the repertoire of IgG-secreted antibody responses by testing against different specific antigens targeting immunization with polysaccharides from a bacterial capsule (pneumococcal polysaccharide, PCP) and inactivated toxin (Tetanus toxoid, T.tox) (Figure 4D–E). All CVID patients and HCs exhibited IgG antibodies against PCP with similar levels for condition I stimulation and slightly elevated levels after condition II stimulation in the HCs compared to the CVID patients (Figure 4D). Anti-T.tox IgG ELISA revealed significantly higher anti-T.tox IgG antibody levels in the HCs compared to the CVID patients after stimulation II. However, stimulation protocol II was more effective in inducing anti-T-tox antibodies compared to protocol I in the CVID patients (Figure 4E).

In order to assess potential induction of autoimmune activity, anti-dsDNA IgG secretion in cell culture supernatants was analyzed (Figure 4F). Neither HCs nor CVID patients secreted those autoantibodies in response to one of the stimulation protocols, with all measured values falling below the lower assay detection limit of 2.6 IE/mL.

## 4. Discussion

A critical function of the adaptive immune system is the ability of B-cells to produce antigen-specific antibodies and to preserve this response by functional memory formation. To achieve this, the immune response requires intact B-cell function, including B-cell activation, differentiation, and proliferation [22]. The markedly decreased or absent levels of serum immunoglobulins in combination with reduced levels of CS MBCs in CVID patients suggest a critical disruption in this patient group in one or more steps of this process [23].

Several studies indicate that impairments of humoral immunity in CVID patients not only result from B-cell intrinsic defects but may also follow B-cell extrinsic changes of immune cell interactions, such as defects in cytokine signaling or limited T-cell support [11,12,13,14,24]. In a previous publication, we showed that after *in vitro* stimulation, a subset of CVID patients was able to retain MBC capacity to differentiate into ASCs and produce antibodies, although often with impaired expansion and functionality compared to healthy individuals [16].

In the present study, we comparatively analyzed two different *in vitro* stimulation protocols to evaluate functional memory formation and antibody production potential in CVID patients. Protocol I used a synergistic stimulation approach, engaging multiple signaling pathways by involving B-cell receptor (activated by SAC), Toll-like receptor 9 (using CpG), and a general mitogen stimulation (using PWM). This protocol was previously shown to result in robust B-cell expansion, enumerating B-cell counts and allowing the application of further assays like flow cytometry and ELISpot (18).

Additionally, we established a second protocol employing a combination of IL-21, CD40L, and CpG to optimally promote B-cell differentiation and activation in a more T-cell-driven manner. In this approach, CD40L mimics T-cell help by engaging the CD40 receptor, which is essential for B-cell survival, activation, and proliferation [25]. IL-21 synergizes with CD40L to facilitate B-cell proliferation and differentiation while promoting class-switch recombination (CSR) through the activation of downstream signaling pathways, including the JAK/STAT pathway, and the induction of activation-induced cytidine deaminase (AID) [26]. Additionally, CpG activates B-cells via Toll-like receptor 9 (TLR9) [27]. This protocol offers advantages over traditional methods by leveraging multiple pathways involved in B-cell activation and differentiation, mimicking more closely the physiological conditions encountered during immune responses and influencing the potential of secreting class-switched immunoglobulins (IgG and IgA).

We observed that B-cells from all CVID patients were able to differentiate into ASCs and to secrete IgG and IgM after receiving sufficient supply during *in vitro* cultivation, even though the patients’ B-cells were severely restricted in their antibody production *in vivo*. Moreover, although the patients had significantly lower frequencies of CS MBC *ex vivo*, the capacity of MBCs to differentiate and proliferate into IgG/IgM secreting cells was comparable to those of the HCs. Our data indicate that *in vitro* stimulation of MBCs for differentiation into ASCs is possible in both protocols in CVID patients with a better response to the more T-cell-help-driven protocol II.

The two protocols differ significantly with regard to the potential stimulation targets. The first protocol is thought to lead to a more general and transient state of B-cell activation, without offering more specific signals that are required for CSR and effective and sustained differentiation into MBCs or long-lived PBs. Being regarded as a mostly T-cell independent activation, stimulation used in protocol I is conversely expected to result in short-lived and moderate affinity maturation of antibody response [22,28]. Protocol II, including CD40L and IL-21, is likely to induce a more physiologically relevant stimulation of B-cell differentiation and enhance CSR and affinity maturation [29]. However, it remains unclear as to what extent the improved antibody secretion might result from an overall better proliferative response to protocol II.

CVID is a heterogeneous immunodeficiency, and only a minority of CVID cases are caused by monogenetic defects [7]. Immunophenotypic characterization, therefore, remains the cornerstone of diagnosis as well as classification. Apart from genetics, immunophenotyping and its resulting classifications are the principal components used to identify potential associations between paraclinical and clinical findings [18]. However, immunophenotyping does not address the functional potential of the B-cell compartment. Unfortunately, labor-intensive and costly protocols remain relevant limiting factors for a broader application. An alternative approach might be the analysis of transcription factors involved in B-cell proliferation and plasma cell differentiation. Indeed, *in vitro* stimulation using anti-IgM, CpG, IL-21, and sCD40L was reported to induce plasma cell differentiation in CVID patients, and flow cytometry revealed the induction of CD38^+^IRF4^+^BLIMP-1^+^ cells [30]. Evaluating transcription factors in combination with the functional ELISpot analysis applied here could potentially enable the development of an easier, accessible functional assay for CVID patients.

Consistent with this approach, Marasco et al. reported B-cell activation responses to either CD40L, as T-cell-dependent stimulus, or CpG, as T-cell-independent stimulus of TLR9, in CVID patients as well as in patients with selective IgA deficiency using flow cytometry and checking upregulation of transcription factors for plasma cell differentiation (*PRDM1*, encoding BLIMP-1) as well as for class switching and somatic hypermutation (AID). Their data support the combination of CpG and CD40L in order to achieve maximal proliferation and differentiation effects [31].

Linking clinical data and functional analysis of B-cells, Desjardins et al. reported that CVID patients not responding to *in vitro* stimulation with IL-4 and IL-21 expressed a significantly higher frequency of non-infectious complications [32]. Since all patients in our cohort responded, we were not able to statistically reproduce this observation.

A limitation of our study is the relatively small sample size and the older age of the participating CVID patients. Our results require replication in a larger cohort covering a wider age range. Another limitation is that our analysis of circulating B-cells from peripheral blood may not fully represent the immunological processes occurring within secondary lymphoid organs, such as in germinal centers of lymph nodes, or at mucosal surfaces. Moreover, considering the heterogeneity of alterations in the B-cell compartment of CVID patients, the methodology used here has important limitations. Future studies should involve a more differentiated approach by analyzing the *in vitro* potential of different B-cell subsets. In addition, evaluating the regulation of transcription factors such as BLIMP-1, XBP1, and IRF4 could help address B-cell aspects of plasma cell differentiation and antibody secretion.

To the best of our knowledge, this is the first study evaluating antibody specificities after *in vitro* stimulation of B-cells from CVID patients. We were able to detect IgG antibodies specific against tetanus toxoid and *Streptococcus pneumoniae* in supernatants after stimulation with both protocols. The overall response was better after protocol II, which is closer to mimicking T-cell-dependent stimulation. Although MBC ELISpot assays, detecting ASCs attained from reactivated MBCs during stimulation culture, are a validated methodology [19], newer methodologies, including sorting of targeted B-cell populations prior to stimulation, would allow a much higher granularity in understanding the differentiation process.

Precise mechanisms of improved *in vitro* antibody secretion following stimulation that mimics T-cell help remain to be established. In addition to compensating for missing T-cell stimulation (B-cell extrinsic), it is possible that the effect of supraphysiologic stimulation may affect the survival of CD27^+^ B-cells by restoring Bcl-family protein imbalance (B-cell intrinsic) [33].

Our data show that a functional restoration of antibody secretion is possible; however, following *in vitro* stimulation, CVID patients maintain significantly lower absolute cell numbers of antibody-secreting plasmablasts compared to healthy controls. Future studies should include a broader range of antibody specificities and extend beyond mere detection through ELISA, incorporating additional assessments of antibody avidity or, where applicable, neutralization capacity.

The absence of dsDNA autoantibodies after *in vitro* expansion is a promising first observation; however, a more in-depth analysis of autoantibodies will be required to address the potential contribution of *in vitro* B-cell stimulation in CVID patients to autoantibody formation.

Differentiation into IgA-secreting cells remained significantly impaired in CVID patients under both *in vitro* stimulation protocols. Future B-cell regenerative strategies should continue to optimize the protocol to achieve an improved IgA switch and secretion of IgA antibodies, since current immunoglobulin replacement therapy does not substitute for the lack of IgA. Data on the combined use of IL-21, IL-4, and CD40L were shown to induce IgA secretion in CVID patients [34]. IL-21 was also reported to positively affect altered apoptosis regulation in CVID B-cells [33,34]. However, it was also shown that IL-4 is only beneficial for B-cell differentiation together with low CD40L stimulation [35], while continuous IL-4 stimulation might even inhibit immunoglobulin secretion and hamper PB differentiation [29]. It remains to be confirmed whether IgA secreted after *in vitro* stimulation would be restrained to the circulation in peripheral blood or could also reach mucosal surfaces.

A possible benefit of protocol II, applied here, is that these reagents are available in clinical-grade quality, thereby fulfilling one of the requirements of Good Clinical Practice (GCP) in future applications.

Given the heterogeneity in CVID patients, it is to be expected that not all patients will respond uniformly to stimulation protocols. Assessing B-cell proliferation and differentiation potential in CVID *in vitro* under different stimulation conditions may help identify novel pathomechanisms and deepen our understanding of B-cell dysfunction in general. Moreover, this methodology is well-suited for evaluating functional MBC response in this patient population to specific antigens, such as those encountered after vaccination [36,37].

In summary, B-cell differentiation capacity and IgG secretion following T-cell dependent *in vitro* stimulation was observed in the majority of CVID patients. In addition, analysis of antibody specificity revealed secretion of immunoglobulins against tetanus and *Streptococcus pneumoniae*, while no double-stranded DNA auto-antibodies were detectable. The applied *in vitro* stimulation protocols failed to achieve sufficient IgA secretion. Future efforts should develop tailored stimulation protocols to restore MBC formation, which could inform strategies for *in vivo* restoration and targeted therapies.

## Figures and Tables

**Table 1:** Patient demographics and immunological data.

Patient	Age	Sex	IgG	IgA	IgM	CD4+	Anti-PCP Antibody Vaccine Response	CD8+	CD19+	CS MBC	NK Cells	Neutrophils
1	29	F	1.98	0.06	0.05	0.49	no response	0.69	0.19	0.820	0.2	3.2
2	34	M	0.71	0.06	0.06	0.68	no response	0.24	0.06	0.010	0.06	2.5
3	37	F	1.90	0.11	0.12	0.33	no response	0.34	0.07	0.030	0.07	1.8
4	56	F	1.50	0.06	0.3	0.45	n.a.	0.17	0.09	0.960	0.33	3.7
5	31	F	1.00	0.06	0.1	0.74	no response	0.21	0.06	0.010	0.05	5.1
6	59	F	0.30	0.06	0.05	0.57	n.a.	0.38	0.01	0.000	0.08	2.0
7	53	F	0.40	0.08	0.38	2.36	n.a.	3.5	1.5	0.000	0.99	7.5
8	55	M	0.30	0.06	0.1	0.6	no response	0.9	0.14	0.030	0.08	3.2
9	51	M	0.30	0.06	0.05	0.64	n.a.	0.62	0.04	0.030	0.05	7.5
10	62	F	2.76	0.16	0.05	0.57	no response	0.32	0.07	1.190	0.11	2.9
11	47	M	1.80	0.06	0.12	1.47	no response	0.84	0.25	0.470	0.04	4.1
12	60	M	1.69	0.06	0.28	0.53	no response	0.62	0.21	1.320	0.14	5.7
13	57	M	5.17	0.06	0.05	0.29	n.a.	0.2	0.07	0.000	0.04	3.6
median	53		1.5	0.06	0.1	0.57		0.38	0.07	0.03	0.08	3.6

Immunoglobulin levels (IgG, IgA, IgM) in g/L before replacement therapy. Class-switched memory B-cells (CS MBCs) as % of CD19^+^ B-cells. All other cell counts are shown per nL. F: female; M: male; n.a.: not assessed; NK cells: natural killer cells.

**Table 2:** Patients’ clinical data and genetics.

Patient	ITP	GLILD	Splenomegaly	Bronchiectasis	Rec. Pneumonia	Rec. Sinusitis	Genetics
1	No	No	No	No	No	Yes	negative
2	No	Yes	Yes	Yes	No	No	negative
3	Yes	Yes	Yes	No	No	Yes	negative
4	Yes	Yes	Yes	No	No	Yes	VUS in PIK3CD
5	Yes	Yes	Yes	No	Yes	Yes	negative
6	No	Yes	Yes	No	Yes	Yes	negative
7	No	Yes	Yes	No	Yes	No	negative
8	No	Yes	No	No	No	No	negative
9	No	No	Yes	Yes	Yes	Yes	negative
10	No	Yes	Yes	Yes	Yes	Yes	negative
11	Yes	Yes	Yes	No	No	No	negative
12	No	No	No	No	No	No	VUS in IRF2BP2
13	No	No	Yes	No	Yes	Yes	negative

GLILD: granulomatous lymphocytic interstitial lung disease; IRF2BP2: interferon regulatory factor 2 binding protein 2; ITP: immune thrombocytopenia; PIK3CD: Phosphatidylinositol-4,5-Bisphosphate 3-Kinase Catalytic Subunit Delta; Rec.: recurrent; VUS: variant of unknown significance.

## Data Availability

The raw data supporting the conclusions of this article will be made available by the authors on request.

## Supplementary Materials

The following supporting information can be downloaded at https://www.mdpi.com/article/10.3390/jcm14207246/s1: Figure S1: Representative flow cytometry gating strategy for the identification of B-cell subsets from human peripheral blood mononuclear cells (PBMC) of a healthy control; Table S1: Surface markers for B-cell phenotyping; Table S2: B-cell subsets within single, living CD3^−^CD19^+^ lymphocytes.

## Abbreviations

The following abbreviations are used in this manuscript:

AEC	3-Amino-9-ethyl-carbazole
AID	Activation-induced cytidine deaminase
ASC	Antibody-secreting cell
BCR	B-cell receptor
BLIMP-1	B lymphocyte-induced maturation protein-1
BSA	Bovine serum albumin
CD40L	CD40 ligand
CPG	Class B CpG oligonucleotide-stimulatory human TLR9 ligand
CS	Class-switched
CSR	Class-switch recombination
CVID	Common variable immunodeficiency disorder
DMF	Dimethylformamide
dsDNA	Double-stranded desoxyribonucleic acid
ELISA	Enzyme-linked Immunosorbent assay
ELISpot	Enzyme-linked immunosorbent spot
ESID	European Society for Immunodeficiencies
FACS	Fluorescence activated cell sorting
FCS	Fetal calf serum
GC	Germinal center
GCP	Good clinical practice
GLILD	Granulomatous lymphocytic interstitial lung disease
HC	Healthy control
IEI	Inborn errors of immunity
IL-21	Interleukin 21
ITP	Immune thrombocytopenia
IQR	Interquartile range
MBC	Memory B-cell
NGS	Next-generation sequencing
ON	Overnight
PB	Plasmablast
PBMC	Peripheral blood mononuclear cell
PCP	Pneumococcal capsular polysaccharide
PRDM1	PR domain zinc finger protein 1
PWM	Pokweed mitogen
Rec.	Recurrent
RT	Room temperature
SAC	Staphylococcus aureus Cowan
SHM	Somatic hypermutation
T.Tox	Tetanus toxoid
TLR9	Toll-like receptor 9
WES	Whole exome sequencing
β-ME	β-Mercaptoethanol
