# Supplementary material for: Antibody Secretion Capacity in CVID Patients: Immunoglobulin Isotypes and Antigen Specificities After T-Cell-Dependent In Vitro Stimulation
Source: J Clin Med. 2025 Oct 14;14(20):7246. doi: 10.3390/jcm14207246 (PMC12565346; doi:10.3390/jcm14207246)
Supplement: Supplementary file 1 [file jcm-14-07246-s001.zip › jcm-3866923-supplementary.pdf]

Journal of
Clinical Medicine

MDPI

Table S1: Surface markers for B-cell phenotyping

Target	Conjugate	Clone	Company	Cat. No.	Dilution
		FACS Antibodies			
Human CD3	PB	UCHT1	Biolegend	300417	1:50
Human CD19	PE/Cy7	HIB19	Biolegend	302216	1:66
Human CD21	PE	Bu32	Biolegend	354904	1:50
Human CD24	PerCp/Cy5.5	ML5	Biolegend	311116	1:66
Human CD27	FITC	M-T271	Biolegend	356404	1:40
Human CD38	AF700	HIT2	Biolegend	303524	1:50
Human IgM	APC	MHM-88	Biolegend	314510	1:40
Human IgD	APC/Cy7	IA6-2	Biolegend	348218	1:50
		Live/Dead discrimination			
Live/Dead Fixable Aqua	PO		Thermo Fisher	L34957	1:50

Table S2: B-cell subsets within single, living CD3$^-$CD19$^+$ lymphocytes

B-cell subset	Marker
Exhausted B-cells	CD21^{low} CD38^{low}
Naïve B-cells	IgD$^+$CD27$^-$
Marginal zone like B-cells	IgD$^+$CD27$^+$
Transitional B-cells	IgD$^+$CD27$^-$CD38^{++}CD24^{++}
Memory B-cells	IgD$^-$CD27$^+$
IgM only memory B-cells	IgD$^-$CD27$^+$IgM$^+$
Class-switched memory B-cells	IgD$^-$CD27$^+$IgM$^-$
Plasmablasts	CD38^{++}CD27^{++}IgD$^-$
IgM only plasmablasts	CD38^{++}CD27^{++}IgD$^-$IgM$^+$
Class-switched plasmablasts	CD38^{++}CD27^{++}IgD$^-$IgM$^-$

 https://doi.org/10.3390/jcm14207246

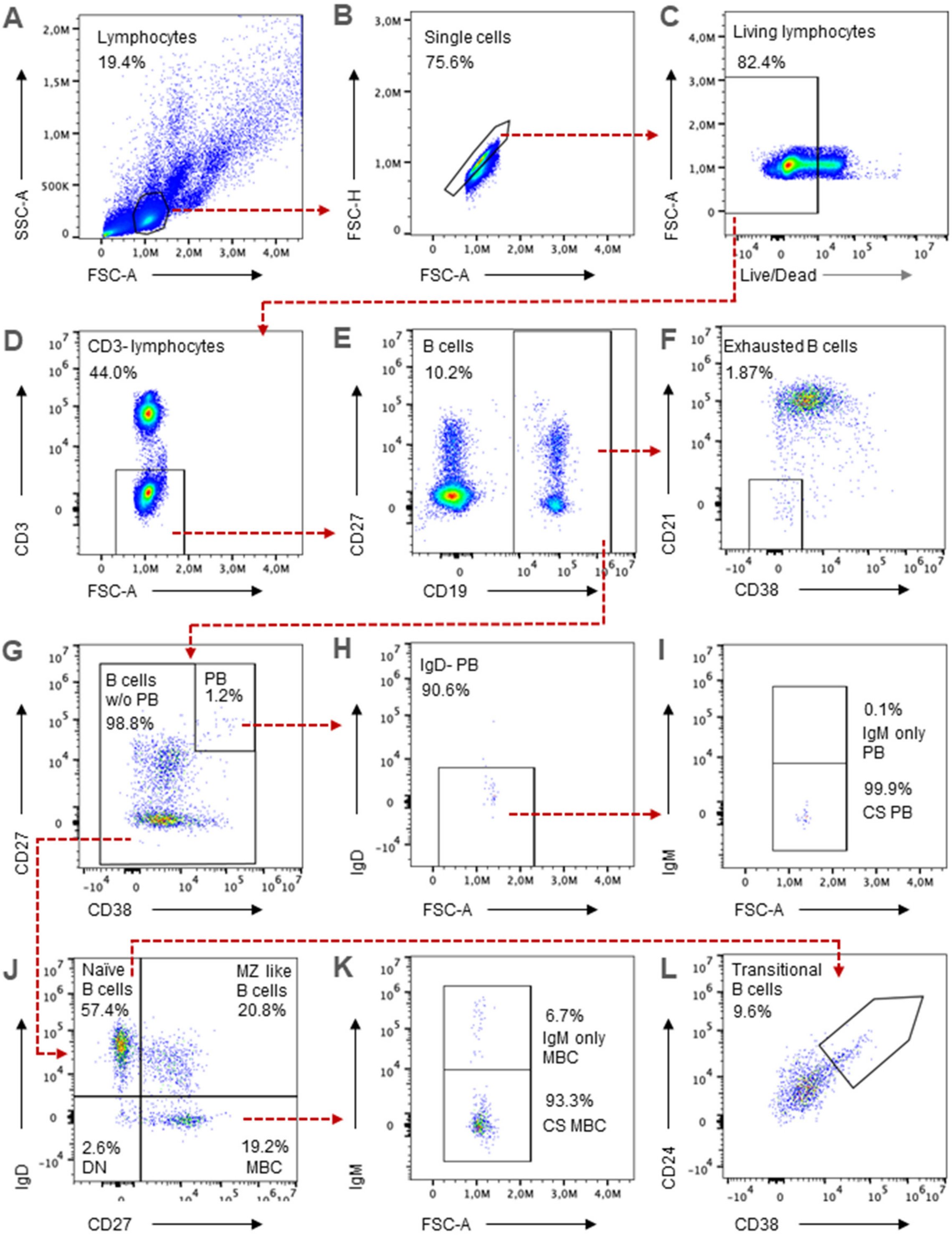

Figure S1: Representative flow cytometry gating strategy for the identification of B-cell subsets from human peripheral blood mononuclear cells (PBMC) of a healthy control. **(A)** Lymphocytes are first selected based on SSC-A and FSC-A. **(B)** Single cells are then gated using FSC-A versus FSC-H, followed by **(C)** the exclusion of dead cells using a Live/Dead stain to isolate viable lymphocytes. **(D)** CD3- non-T-cells are selected, and **(E)** B-cells are identified as $CD19^{+}$. **(F)** Exhausted B-cells are further gated as $CD21^{low}$ $CD38^{high}$. Subsequent gating focuses on the identification of **(G)** plasmablasts (PB), defined as $CD38^{++}CD27^{++}$ cells, further gated as **(H)** $CD38^{++}CD27^{++}$IgD- and subsequently gated for IgM expression **(I)** to discriminate class-switched (CS) $CD38^{++}CD27^{++}IgD^{-}IgM^{-}$ and IgM only $CD38^{++}CD27^{++}IgD^{-}IgM^{+}$ PB. Remaining B-cells without

(w/o) PB were gated for **(J)** naïve B-cells IgD^+ $CD27^-$, marginal zone (MZ)-like B-cells ($CD27^+$ IgD^+), and memory B-cells (MBC; IgD^- $CD27^+$) of which MBC were further stratified regarding IgM expression into **(K)** CS IgD^-CD27^+IgM- and IgM only $IgD^-CD27^+IgM^+$ MBC. **(L)** Transitional B-cells are identified as IgD^+CD27^- $CD38^{++}CD24^{++}$.
